# Protection against LPS-induced cartilage inflammation and degradation provided by a biological extract of *Mentha spicata*

**DOI:** 10.1186/1472-6882-10-19

**Published:** 2010-05-11

**Authors:** Wendy Pearson, Ronald S Fletcher, Laima S Kott, Mark B Hurtig

**Affiliations:** 1 Dept Plant Agriculture, University of Guelph, Guelph Ontario, Canada; 2 Department of Clinical Studies, University of Guelph, Guelph Ontario, Canada

## Abstract

**Background:**

A variety of mint [*Mentha spicata*] has been bred which over-expresses Rosmarinic acid (RA) by approximately 20-fold. RA has demonstrated significant anti-inflammatory activity *in vitro *and in small rodents; thus it was hypothesized that this plant would demonstrate significant anti-inflammatory activity *in vitro*. The objectives of this study were: a) to develop an *in vitro *extraction procedure which mimics digestion and hepatic metabolism, b) to compare anti-inflammatory properties of High-Rosmarinic-Acid *Mentha spicata *(HRAM) with wild-type control *M. spicata *(CM), and c) to quantify the relative contributions of RA and three of its hepatic metabolites [ferulic acid (FA), caffeic acid (CA), coumaric acid (CO)] to anti-inflammatory activity of HRAM.

**Methods:**

HRAM and CM were incubated in simulated gastric and intestinal fluid, liver microsomes (from male rat) and NADPH. Concentrations of RA, CA, CO, and FA in simulated digest of HRAM (HRAM_sim_) and CM (CM_sim_) were determined (HPLC) and compared with concentrations in aqueous extracts of HRAM and CM. Cartilage explants (porcine) were cultured with LPS (0 or 3 μg/mL) and test article [HRAM_sim _(0, 8, 40, 80, 240, or 400 μg/mL), or CM_sim _(0, 1, 5 or 10 mg/mL), or RA (0.640 μg/mL), or CA (0.384 μg/mL), or CO (0.057 μg/mL) or FA (0.038 μg/mL)] for 96 h. Media samples were analyzed for prostaglandin E_2 _(PGE_2_), interleukin 1β (IL-1), glycosaminoglycan (GAG), nitric oxide (NO) and cell viability (differential live-dead cell staining).

**Results:**

RA concentration of HRAM_sim _and CM_sim _was 49.3 and 0.4 μg/mL, respectively. CA, FA and CO were identified in HRAM_sim _but not in aqueous extract of HRAM. HRAM_sim _(≥ 8 μg/mL) inhibited LPS-induced PGE_2 _and NO; HRAM_sim _(≥ 80 μg/mL) inhibited LPS-induced GAG release. RA inhibited LPS-induced GAG release. No anti-inflammatory or chondroprotective effects of RA metabolites on cartilage explants were identified.

**Conclusions:**

Our biological extraction procedure produces a substance which is similar in composition to post-hepatic products. HRAM_sim _is an effective inhibitor of LPS-induced inflammation in cartilage explants, and effects are primarily independent of RA. Further research is needed to identify bioactive phytochemical(s) in HRAM_sim_.

## Background

Rosmarinic acid (RA; C_18_H_16_O_8_) is a polyphenolic carboxylic acid found in many herbal plants including rosemary (*Rosmarinus officinalis*), oregano (*Origanum vulgare*) and mint (commonly *Mentha spicata *or *Mentha × piperita*). RA has widely reported biological activities in mammals and mammalian cells including antioxidant [1], anti-inflammatory [2], antitumor [3,4], immunomodulatory [5], antiviral [4] and antibacterial [6]. There is considerable scientific support for an anti-inflammatory role for RA. It has shown significant inhibitory effects on inflammation induced by lipopolysaccharide (LPS) in bone-marrow-derived dendritic cells [7], primarily by inhibiting chemokine recruitment of macrophages via the Mitogen Activated Protein Kinase (MAPK) cell signalling pathway. RA has also shown inhibitory effect on LPS-induced production of nitric oxide (NO) and inducible nitric oxide synthase (iNOS) in macrophages, an action mediated in part by an ability of RA to prevent phosphorylation of an inhibitor protein on NF-κB (Iκ-Bα). This prevents binding of this nuclear transcription factor to DNA encoding a series of inflammatory proteins, thus reducing their biological expression [8].

Mint (*Mentha spicata*) is a common natural source for RA. Like pure RA, mint oil also inhibits the inflammatory consequences of LPS, including inhibition of interleukin-1 (IL-1), prostaglandin E_2 _(PGE_2_), leukotriene B_4 _(LTB_4_) production by LPS-stimulated human monocytes [9]. As these biological actions are considered to be related to the RA content of the plant, considerable effort has been invested in developing strategies to upregulate biosynthesis of RA by genetically modified (GMO) plant tissues [10,11]. These efforts have successfully resulted in RA production of up to 45 mg/g plant tissue (dry weight; DW). However, widespread commercialization of these technologies has lagged, due in varying degrees to technical difficulty, low capacity for production of biomass, and complex regulatory environment for GMO products. Thus, there remains a commercial opportunity for agronomic selection of plants with naturally robust biosynthesis of RA for the nutraceutical and biotechnology markets.

Recently, selective breeding of *Mentha spicata *clones has generated plants which naturally over-produce RA, resulting in tissue concentrations of up to 122 mg/g DW [12,13] - more than double the content of high-RA-producing control clones and three times higher than other GMO plants [10]. The processed High-Rosmarinic-Acid *M. spicata *resulting from these experiments (HRAM) has shown marked antioxidant activity *in vitro *[12,13] and may be an ideal candidate for nutritional intervention for inflammatory diseases.

We have previously described an *in vitro *cartilage explant model to assess the cartilage-sparing and anti-inflammatory properties of dietary nutraceuticals [14,15]. While this model effectively accounts for the effects of gastric digestion and ultrafiltration of molecules, it does not provide any measure of biotransformation/bioactivation which occurs in the liver *in vivo*. This is a significant limitation when assessing the bioactivity of herbal products *in vitro *because secondary plant metabolites are, in many cases, extensively modified by cytochrome P450 enzymes [16,17]. Thus, observed bioactivity of secondary plant metabolites (such as rosmarinic acid) *in vitro *may not accurately reflect their bioactivity *in vivo*.

The objectives of the current study were 1) to produce a biological extract of HRAM by adapting an artificial digestion procedure for the purpose of simulating hepatic metabolism of putative anti-inflammatory botanicals, 2) to compare the anti-inflammatory and/or chondroprotective properties of HRAM and a wild-type *M. spicata *(CM) in LPS-stimulated cartilage explants, and 3) to determine whether anti-inflammatory activity of HRAM can be attributed to its RA content, or the primary hepatic metabolites of RA.

We hypothesized that 1) a biological extraction procedure which simulates digestion and hepatic metabolism of HRAM (HRAM_sim_) would produce a substance containing RA and its primary hepatic metabolites including caffeic acid (CA), *m*-coumaric acid (CO), and ferulic acid (FA) [18]; 2) HRAM_sim _would modulate the inflammation and degradation associated with exposing cartilage explants to LPS to a greater magnitude than CM; and 3) anti-inflammatory activity of HRAM_sim _would be attributed, at least in part, to RA and/or hepatic metabolites of RA.

## Methods

All materials and reagents were purchased from Sigma Aldrich (Mississauga ON Canada) unless otherwise stated.

### Plant material

Spearmint seed (*Mentha spicata *L.) was sourced from Stokes Seeds Ltd., St. Catharines ON, Canada. Seed was germinated at the University of Guelph (Dept of Plant Agriculture) and planted in a research plot at the University of Guelph. Plant material was harvested at vegetative maturity and air dried at 35°C to a dry matter content of ~89%.

#### Simulated digestion and hepatic metabolism

HRAM and CM (0.85 g) were individually added to aliquots of simulated gastric fluid and simulated intestinal fluid as previously described [14,19,20]. Resulting mixtures were filtered (0.22 μm) and pH was adjusted to 7.4. In order to simulate hepatic metabolism of RA, liver microsomes from rat (male) (Sigma Aldrich, Mississauga ON Canada) were added to a final concentration of 0.03 mg/mL [21] followed by NADPH (100 μg in 0.01 M NaOH) [21]. Solutions were incubated for a further 30 min at 37°C, 7% CO_2_[21], followed by centrifugation (2500 rpm for 20 min). Supernatants were filtered (0.22 μm), placed into 50 kDa ultrafiltration centrifuge units (Amicon Ultra; Millipore, Mississauga ON), and centrifuged at 3000 rpm for 25 min at room temperature. The resulting 50 kDa fractions were refrigerated (4°C) until use. A 'blank' simulated digest was made using the identical methodology but without including any HRAM or CM.

### Phytochemical analysis of simulated digests

Metabolic breakdown products of RA were quantified by HPLC. Briefly, a Gilson 506C HPLC system equipped with Unipoint 2.1 software (Gilson, Middleton, WI, USA) equipped with a 234 auto-injector, dual pumps, column heater, and 118 UV/Vis detector was used for metabolite detection. Digests were injected onto a Supelco C18 Discovery column (250 × 4.6 mm, 35°C, detector sensitivity 0.015) and eluted with a mixture of 0.1% phosphoric acid (A) and acetonitrile (B). Separation was achieved at a flow rate of 1 mL/min using a linear gradient of 83%-77% A over 45 min, then 77-64% A over 2 min, 64-44% A over 10 min, 44-0% over 2 min, maintaining for 4 min, then returning to starting conditions and maintain for 5 min for a total run time of 75 min. Standards of RA (25 μg/mL), CO (5 μg/mL), FA (1 μg/mL), and CA (1 μg/mL) were injected as internal standards.

### Cartilage explants

Cartilage tissue was obtained from a commercial, federally inspected meat packing facility. Cartilage explants (4 mm diameter; 11-17 mg/explant) were excised from the articulating surface of the intercarpal joint of healthy pigs using a sterile dermal biopsy tool [14]. Explants were placed 2 per well into a 24-well tissue culture plate and maintained in culture for a total of 96 h. Media (1000 μL) was removed from each well every 24 h. For the first 48 h of culture (prior to exposure to LPS), media samples were discarded. During the final 48 h (immediately prior to and during stimulation with LPS) samples were collected into sterile microcentrifuge tubes containing 10 μg indomethacin in DMSO and immediately frozen (-20°C) until analysis.

### Treatments

Tissue from a total of 17 animals were used for these experiments. For the first 24 h of culture, tissue culture media [TCM: Dubelco's Modified Eagle Medium plus ascorbic acid, antibiotics (10 mL/L containing 10,000 units penicillin; 10 mg streptomycin/mL), dexamethasone, amphotericin B, amino acids, manganese sulphate, lactalbumin hydrolysate, sodium selenite, NaHCO_3_, and fetal bovine serum (10%)] [19] contained no HRAM_sim _or LPS. From 24 - 120 h of culture, explants were exposed to treatments as follows:

HRAM_sim _[0 (ie. 'blank'), 8, 40, 80 μg/mL] (n = 6)

HRAM_sim _(0, 80, 240, 400 μg/mL) (n = 5)

Assuming a total body water content of 42 L [31], these amounts are approximately equivalent to a daily dose of 0, 0.3, 1.7, 3.3, 10.1 and 16.8 g (respectively) of HRAM for an average 78 kg person. Each treatment was evaluated in the presence or absence of LPS (3 μg/mL).

For experiments in which individual bioactivity of RA, CA, CO, and FA was determined, stock solutions of each compound were prepared using 35% ethanol in distilled water. Aliquots of stock solutions were diluted 1:999 with distilled water. Aliquots of diluted stock solutions were suspended in 'blank' simulated digest and filtered (0.22 μm) before use.

CM (0, 1.0, 5.0, 10.0 mg/mL) (n = 6 - same animals as RA and CO treatments)

RA (0.640 μg/mL), CA (0.384 μg/mL) (n = 6 - same animals as CM and CO treatments)

CO (0.057 μg/mL), or FA (0.038 μg/mL) (n = 6 - same animals as CM and RA treatments)

As the RA concentration of HRAM_sim _was approximately 125 times higher than that of CM_sim_, the concentration of CM_sim _used in this experiment was 125 times those of HRAM_sim _in order to standardize the amount of RA to which explants were exposed. Concentration of RA and its metabolites used in this experiment was equivalent to the concentration of each compound in a HRAM_sim _dose of 80 μg/mL (see Table 1)

**Table 1:** Concentrations of metabolites identified in aqueous extract and simulated digest of HRAM and CM.

Compound	HRAM [μg/mL]		Control Mint [μg/mL]	
	
	Aqueous/Ethanolic extract^^^	Simulated digest	Aqueous/Ethanolic extract^^^	Simulated digest
Rosmarinic acid [RA]	720.0	49.3	16.1	0.4

Caffeic acid [CA]	nd*	33.0	2.1	2.2

Ferulic acid [FA]	nd*	3.4	2.5	2.6

Coumaric acid [CO]	nd*	4.6	2.2	2.2

^^^Aqueous/ethanolic extracts are based on 5 mg of leaf tissue in 3 mL of water/ethanol [50:50 v/v], microwave extraction for 2.2 minutes at 240 watts. * not detected

For the final 48 h, all explants were exposed to an inflammatory stimulus (LPS; 0 or 3 μg/mL) in order to upregulate production of inflammatory eicosanoids and catabolic enzymes.

### Sample analysis

All assays plates were read on an ELX 800 Universal Microplate Reader (Biotech Instruments Inc., Winooski, VT) unless otherwise indicated. PGE_2_, GAG, and NO concentrations were determined as follows:

#### PGE_2_

TCM samples were analyzed for PGE_2 _using a commercially available kit (R&D Systems). Plates were read at absorbance of 450 nm. A best-fit 3^rd ^order polynomial standard curve was developed for each plate (*R*^2 ^≥ 0.99), and these equations were used to calculate PGE_2 _concentrations for samples from each plate.

#### GAG

TCM GAG concentration was determined using a 1,9-Dimethyl Methylene Blue (1,9-DMB) spectrophotometric assay [19]. Samples were added to 96-well plates at 50% dilution, and serially diluted 1:2 up to a final dilution of 1:64. Guanidine hydrochloride (275 mg/mL) was added to each well followed immediately by addition of 150 μL DMB reagent. Absorbance was measured at 530 nm. Sample absorbance was compared to that of a bovine chondroitin sulfate standard (Sigma, Oakville ON). A best-fit linear standard curve was developed for each plate (*R*^2 ^≥ 0.99), and these equations used to calculate GAG concentrations for samples on each plate.

#### NO_2_

Nitrite (NO^2-^), a stable oxidation product of NO, was analyzed by the Griess reaction [19]. Undiluted TCM samples were added to 96 well plates. Sulfanilamide (0.01 g/mL) and N-(1)-Napthylethylene diamine hydrochloride (1 mg/mL) dissolved in phosphoric acid (0.085 g/L) was added to all wells, and absorbance was read within 5 min at 530 nm. Sample absorbance was compared to a sodium nitrite standard. A best-fit linear standard curve was developed for each plate (*R*^2 ^≥ 0.99), and these equations were used to calculate nitrite concentrations for samples from each plate.

#### Cell viability

Viability of cells within cartilage explants [all treatments excluding HRAM (240 and 400 μg/mL) was determined using a Calcein-AM (C-AM)/Ethidium homodimer-1 (EthD-1) cytotoxicity assay kit (Molecular Probes) modified for use in cartilage explants [19]. C-AM and EthD-1 were mixed in sterile distilled water at concentrations of 4 and 8 μM, respectively. Explants were placed one per well into a sterile 96-well microtitre plate and incubated in 200 μL of the C-AM/EthD-1 solution for 40 min at room temperature. The microplate reader (Victor 3 1420 Microplate Reader, Perkin Elmer, Woodbridge ON) was set to scan each well, beginning at the bottom, using 10 horizontal steps at each of 3 vertical displacements set 0.1 mm apart. C-AM and EthD-1 fluorescence in explants were obtained with using excitation/emission filters of 485/530 nm and 530/685 nm, respectively.

#### IL-1

TCM from explants treated with HRAM (0, 8, 40, 80 μg/mL) were analyzed for IL-1 using a commercially available kit (R&D Systems). Plates were read at absorbance of 450 nm. A best-fit 3^rd ^order polynomial standard curve was developed for each plate (*R*^2 ^≥ 0.99), and these equations were used to calculate IL-1 concentrations for samples from each plate.

### Statistical analysis

Data from the final 48 h of culture (ie. from immediately prior to and during exposure to LPS) are presented as mean ± SE. Time '0 h' is the baseline sample after the first 48 h of culture before addition of LPS. Each animal represents a single observation (ie. experimental unit). To compare effects of treatments over time, PGE_2_, GAG, NO and IL-1 data were analyzed using 2-way ANOVA (SigmaStat, Version 11) with respect to treatment and time. To determine changes in dependent variables over time within individual treatments, one-way ANOVA was used to detect changes in dependent variables over time within treatments. Viability data were analyzed using a paired difference *t*-test comparing each treatment with control. When a significant F-ratio was obtained, the Holm-Sidak post-hoc test was used to detect significantly different means. Significance was accepted when p < 0.05.

## Results

### HPLC analysis

**Figure 1:**
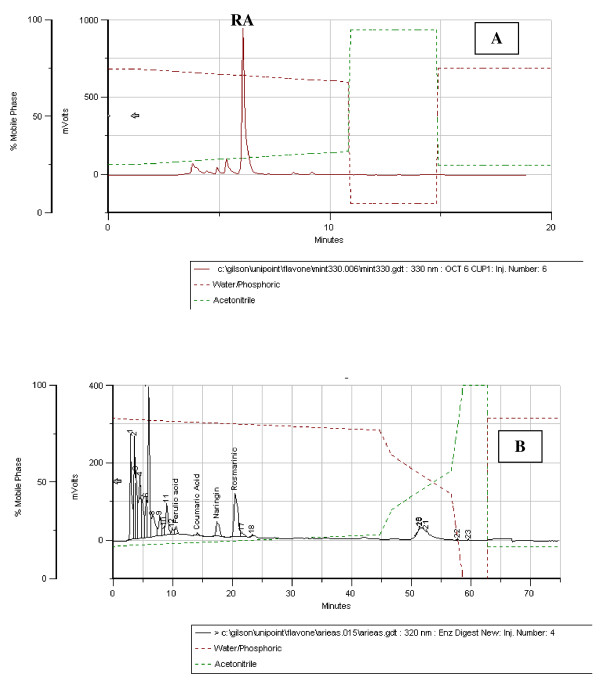
**HPLC chromatogram of aqueous extract [A] and biological extract B] of High-Rosmarinic Acid Mint [HRAM]**. [A] The major peak represents rosmarinic acid [RA] at a concentration of 130 ug/mL. [B] Retention times of compounds of interest: Caffeic acid = 6.00 min; Ferulic acid = 10.52 min; m-Coumaric acid = 14.10 min; Rosmarinic acid = 20.50 min.

Chromatogram of HRAM aqueous extract and HRAM simulated digest is provided in Figure 1A and 1B respectively. Concentrations of RA and its primary hepatic metabolites identified in HRAM_sim_, CM_sim_, and aqueous extracts of HRAM and CM are reported in Table 1.

### PGE_2_

**Figure 2:**
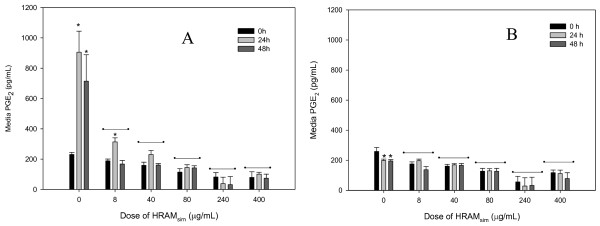
**Figure - Prostaglandin E_2 _[PGE_2_] production by cartilage explants stimulated with LPS [3 or 0 μg/mL; Panels A and B, respectively]**. Explants were conditioned with HRAM_sim _[0, 8, 40, 80, 240 or 400 μg/mL] for 96 h, and were stimulated with LPS for the final 48 h. Data shown are for the final 48 hours only. * represents significant effect of LPS; -- represents treatments significantly different from controls.

LPS significantly increased PGE_2 _in explants which were not conditioned with HRAM_sim _(Figure 2A). Compared with controls, HRAM_sim _induced a strong, dose-dependent inhibition of PGE_2 _at all doses tested. LPS produced a significant increase in PGE_2 _in explants conditioned with the lowest dose of HRAM_sim _(8 μg/mL), but PGE_2 _production by these explants was still significantly lower than in controls. LPS did not increase PGE_2 _production by explants conditioned with all other doses of HRAM_sim_.

In unstimulated explants, HRAM_sim _(8, 40, 80, 240 and 400 μg/mL) significantly reduced PGE_2 _at all time points compared with unstimulated controls (Figure 2B).

There was no significant effect of any dose of CM_sim_, RA or any RA metabolite on PGE_2 _production in LPS-stimulated or unstimulated explants (Additional file 1).

### NO

**Figure 3:**
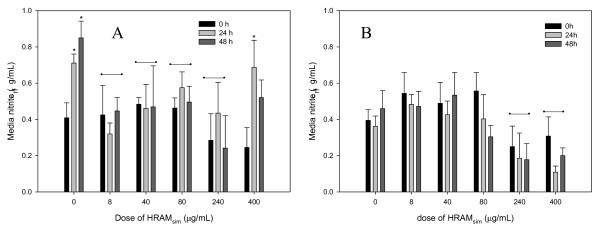
**Nitric Oxide [NO] production by cartilage explants stimulated with LPS [3 or 0 μg/mL; Panels A and B, respectively]**. Explants were conditioned with HRAM_sim _[0, 8, 40, 80, 240 or 400 μg/mL] for 96 h, and were stimulated with LPS for the final 48 h. Data shown are for the final 48 hours only. * represents significant effect of LPS; -- represents treatments significantly different from controls.

LPS significantly (p < 0.001) increased nitric oxide production compared with unstimulated control explants (Figure 3A). All concentrations of HRAM_sim _significantly inhibited NO response to LPS, with the exception of 400 μg/mL, which non-significantly (p = 0.1) reduced NO.

In unstimulated explants, only the highest doses of HRAM_sim _(240 and 400 μg/mL) significantly reduced nitric oxide production (Figure 3B).

There was no significant effect of CM_sim_, RA or any RA metabolites on NO production by LPS-stimulated or unstimulated explants (Additional file 1).

### GAG

**Figure 4:**
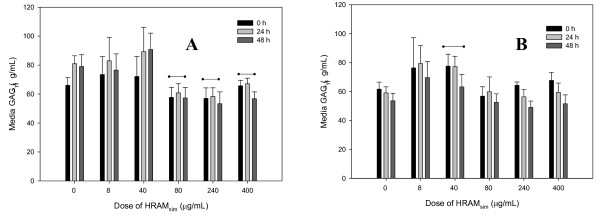
**Glycosaminoglycan [GAG] production by cartilage explants stimulated with LPS [3 or 0 μg/mL; Panels A and B, respectively]**. Explants were conditioned with HRAM_sim _[0, 8, 40, 80, 240 or 400 μg/mL] for 96 h, and were stimulated with LPS for the final 48 h. Data shown are for the final 48 hours only. * represents significant effect of LPS; -- represents treatments significantly different from controls.

LPS significantly (p < 0.001) increased media GAG concentrations compared with unstimulated control explants (Figure 4A and 4B). LPS-induced GAG release was significantly inhibited by HRAM_sim _(80, 240 and 400 μg/mL), but not by the two lowest doses (Figure 4A).

HRAM_sim _(40 μg/mL) significantly (p = 0.007) increased GAG release by unstimulated explants (Figure 4B).

There was no significant effect of CM_sim _or any RA metabolites in LPS-stimulated explants. RA significantly reduced GAG release in LPS-stimulated explants at 24 h compared with stimulated control explants (Additional file 1).

### Cell viability

**Figure 5:**
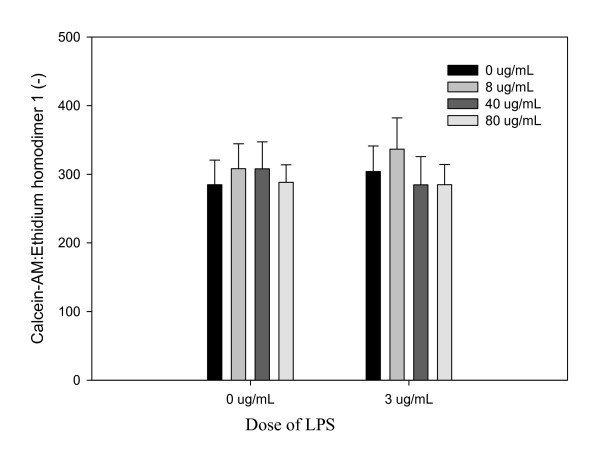
**Viability of cells within cartilage explants stimulated with LPS [0 or 3 μg/mL]**. Explants were conditioned with HRAM_sim _[0, 8, 40, or 80 μg/mL] for 96 h, and were stimulated with LPS [0 or 3 μg/mL] for the final 48 h. Data shown are from the end of the 48 hour stimulation period.

LPS did not significantly alter the ratio of live-to-dead cells within the cartilage explants (Figure 5).

There was no significant effect of HRAM_sim _(Figure 5) or CM_sim _(Additional file 1) on cell viability at any dose either in the presence or absence of LPS.

Viability of unstimulated explants conditioned with CO was significantly reduced (Additional file 1). There were no other significant effects of RA or its metabolites on viability in stimulated or unstimulated explants.

### IL-1

**Figure 6:**
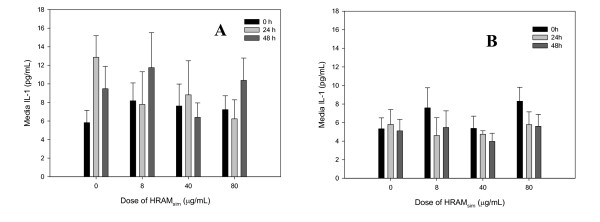
**Interleukin-1β [IL-1] production by cartilage explants stimulated with LPS [3 or 0 μg/mL; Panels A and B, respectively]**. Explants were conditioned with HRAM_sim _[0, 8, 40, or 80 μg/mL] for 96 h, and were stimulated with LPS for the final 48 h. Data shown are for the final 48 hours only.

LPS significantly increased media IL-1 compared with unstimulated controls (p = 0.01) (Figure 6A and 6B).

Peak LPS-induced IL-1 concentration from explants conditioned with HRAM_sim _(80 μg/mL) (6.23 ± 2.0 pg/mL) was approximately 50% that of peak IL-1 in stimulated controls (12.9 ± 2.3 pg/mL), but this difference was not significant (p = 0.1) (Figure 6A).

There was no significant effect of HRAM_sim _conditioning on IL-1 concentration in media from cartilage explants that were not exposed to LPS (Figure 6B).

## Discussion

The current paper describes a simple and effective method for simulating digestion and hepatic metabolism of anti-inflammatory compounds intended for oral administration. We have used this methodology to prepare a biological extract of an herbal plant, which was subsequently demonstrated to have marked inhibitory activity on mediators of cartilage inflammation and degradation.

The biological extraction procedure reported herein is intended to improve upon simulated digestion procedures we have reported previously [14,15] by incorporating the effects of liver metabolism. It is known that many secondary plant metabolites undergo extensive hepatic bioactivation and/or biotransformation [22], and these metabolic end-products often produce biological effects that differ markedly from those of the parent compound [23]. While RA was identified in the aqueous extract of HRAM, no hepatic metabolites were identified in this preparation. Identification of hepatic metabolites in the biological extract provided evidence that incubating HRAM in the presence of liver microsomes and NADPH resulted in metabolism of RA to selected hepatic products, including FA, CA, and CO.

The total amount of RA and its associated hepatic metabolites recovered from the microsomal digest of HRAM was 90.3 μg/mL, representing 12.54% of the RA content of HRAM aqueous/ethanolic extract. This is in reasonable agreement with *in vivo *post-hepatic RA, FA, CA, CO and methyl-RA recoveries reported by others, who report that 5.47% of total RA administered to rats (50 mg/kg BW) is recovered in urine within 18 h after RA administration, either as intact RA or its associated metabolites [18]. This provides evidence that there is considerable disappearance of dietary RA that is not accounted within its known hepatic metabolites (FA, CO, CA and methyl-RA). The relative amounts of RA, CA, FA, and CO identified in urine of rats provided with dietary RA are 1, 0.1, 0.2, and <0.01, respectively. This is in reasonable agreement with the relative contributions of these compounds in our biological extract of 1, 0.6, 0.06, and 0.09, respectively. The differences that are observed between the profile of RA and its metabolites *in vivo *and in our model may result from biotransformation reactions and transporter mechanisms in the kidney; reactions which occur *in vivo *but are not accounted for within our model. Thus, while the biological extraction procedure in the current study provides reasonable approximation of post-hepatic RA metabolism with respect to total RA and metabolite recoveries and composition, there may be benefit to further developing the model to incorporate aspects of renal biotransformation and transport. The fate of remaining 87.4% of RA added to our biological extract is not known; there are a number of peaks on the chromatograph that are, as yet, unidentified. These peaks will be identified as part of our ongoing research.

We observed that CO, CA and FA were identified in the aqueous extract of CM but not in HRAM, owing simply to these compounds accumulating naturally in the variety of mint which we used as our control and not in HRAM. The fact that there was negligible change in these compounds in CM_sim _may be due to differences in the initial amount of RA which was available for metabolism. The RA concentration of CM was markedly lower than in HRAM, and metabolites formed from the metabolism of RA by microsomes in CM_sim _were added to a pre-existing pool of CO, CA and FA. These initial parent phenolics likely endured some level of degradation through the digestion procedure, decreasing their initial concentrations and resulting in a measurement of no net change.

While phytochemical profile of HRAM_sim _in the current study correlates reasonably well with existing literature describing post-hepatic metabolites of RA in rodents, pharmacokinetic (PK) analysis of oral HRAM is necessary to confirm that our biological extraction procedure accurately predicts *in vivo *biotransformation of HRAM. As such, PK studies with HRAM are currently underway in horses and in dogs in our laboratory. There are a number of additional limitations to the current method:

1. There may be species differences in metabolism of secondary plant metabolites. For our experiments we chose male rat microsomes because they were readily available and well-characterized. However, effect of microsomes may differ when they are sources from human, equine or other species.

2. While we were able to identify hepatic metabolites in our biological extract, we did not look for compounds which might preclude a similarity with post-hepatic metabolism, Future studies should incorporate such tests.

3. There are various *in vivo *mechanism such as red cell sequestering of post-hepatic products or compartmentalization of bioactive compounds in certain tissues and/or organs which could make such compounds unavailable for use in inflammatory situations. While it is not reported that such mechanisms act upon RA *in vivo*, it is not known whether they may be a factor in other, as yet unidentified compounds within HRAM. Further studies must attempt to further characterize HRAM with respect to phytochemical profile and attempt to determine *in vivo *storage and/or PK profiles.

The most notable effect of the biological extract of HRAM_sim _in the current study was a dose-dependent and marked inhibitory effect on LPS-induced PGE_2 _by cartilage explants. This was evident even at the lowest dose tested (8 μg/mL), which is approximately equivalent to a dose of 3.3 g for an average 78 kg person (assuming 100% bioavailability of the active constituents). It is not unusual for herbal plants to possess anti-inflammatory properties; indeed, even aspirin has its historical roots in the bark of the white willow tree. What is unique about HRAM, however, is the low dose at which its striking effect on LPS-induced PGE_2 _was observed. To our knowledge, this magnitude of effect has not previously been reported for herbs on cartilage tissue. *M. spicata *is a very robust crop with a long growing season, and can be cultivated in widely divergent agronomic zones making it a potentially important and exciting commercial source for anti-inflammatory products. How HRAM_sim _exerts its effect on LPS-induced PGE_2 _production is not known, but our study did not produce compelling evidence for an important role of RA. We did not identify any significant biological effects of CM, the amount of which was scaled upwards to contain equivalent amounts of RA as in HRAM. Furthermore, neither RA nor three of its hepatic metabolites (CO, CA and FA) demonstrated inhibitory activity towards LPS-induced PGE_2_. Among several candidates for bioactive, methyl-RA may be a significant contributor to the observed anti-inflammatory effect of HRAM_sim _as it is a major hepatic metabolite of RA [18]. We did not test methyl-RA, owing to an inability to obtain reliable standard; the literature does not yet describe anti-inflammatory effects of methyl-RA, either in cartilage or any other tissue, but this is a question that begs investigation in future research.

Given the reported effect of RA [24] and *Mentha sp*. [9] on IL-1 production, we hypothesized that an effect of HRAM_sim _on PGE_2 _would be mediated, at least in part, by an inhibitory effect of HRAM upon LPS-induced IL-1 production. Our data do not support this hypothesis. This may again be a matter of dose, as the amount of RA used in our experiment (0.64 μg/mL) was lower than that reported bioactive by others (1.0 μg/mL) [24]. But it is very interesting that, despite the low concentration of RA in the lowest HRAM dose tested, we still saw marked inhibition of LPS-induced PGE_2 _by HRAM.

The effect of HRAM_sim _on nitric oxide was expected, given the previously demonstrated antioxidant properties of HRAM [13] and *Mentha spicata *[25], as well as reported inhibitory effects of *Mentha sp*. on nitric oxide formation [26,27]. While a clear concentration-dependency in the primary outcome variable (ie. PGE_2_) was observed, all doses of HRAM_sim _performed the same with respect to their ability to inhibit LPS-induced nitric oxide, and indeed there appeared to be a reduction in NO-inhibiting ability at the highest dose of HRAM_sim_. This may simply reflect a circumstance where the maximum threshold of NO inhibition was reached, beyond which exposure of explants to increasing concentrations of HRAM_sim _did not achieve any change in response. Another possible explanation is that HRAM_sim _may inhibit NO production through multiple biological pathways which are sensitive to HRAM_sim _inhibition at varying doses, which could also account for the absence of a net dose-responsive relationship. Searching for answers to this question may involve systematically blocking known contributors to nitric oxide production and observing the inhibitory effect of HRAM_sim _on LPS-induced NO production. It is noteworthy that, contrary to reports that RA also inhibits NO formation [24], our study did not find that RA was effective at reducing LPS-induced nitric oxide. Nor were any of the RA metabolites tested. Thus, we have not identified a causative phytochemical of NO inhibition. The concentration of RA we tested was approximately 64% of doses reported to produce antioxidant activities (1 μg/mL) [24], which may be why we did not see a significant effect of RA on LPS-induced NO. Also, the effect of simulated digestion fluid on bioactivity of RA is not reported, and it is conceivable that this may influence biological activity. Future research should compare RA in simulated digest and RA in physiological saline to determine the effects of vehicle on bioactivity of RA. Assuming that RA (and its associated metabolites) is not the primary bioactive phytochemical, a good candidate for future investigation may be the flavonoid fraction of HRAM. Flavonoids are stable phenolic compounds found abundantly in plants including *Mentha spicata *[25], and have reported modulatory effect on NO formation [28] and NO scavenging [26,27]. While the stability of some flavonoids in the presence of intestinal enzymes has been questioned [29], the flavonoid fraction of HRAM is never-the-less a logical place to continue the search for a primary bioactivity phytochemical in HRAM.

The effect of HRAM on GAG release is a novel finding, and one which has not been previously reported for *Mentha sp*. The mechanism by which HRAM_sim _reduces GAG release is not known, but may be a secondary effect of reduced NO formation, as NO is shown to increases post-translational aggrecan degradation under certain circumstances [30]. Unlike the previous outcome measures discussed, inhibition of LPS-induced GAG release by HRAM_sim _was not independent of RA. RA (0.64 μg/mL) significantly inhibited GAG release at 24 h of LPS exposure. However, peak LPS-induced GAG production at 24 h in RA-conditioned explants (106.2 ± 10.4 μg/mL) was still about 20% higher than peak LPS-induced GAG release from explants conditioned with the lowest dose of HRAM (82.9 ± 16.1 μg/mL), suggesting that other compounds, or synergies between RA and its metabolites, magnify the protective effect of RA on GAG loss. We did not test the combined (synergistic) effect of RA and its metabolites, and this should be tested in future research. Furthermore, future research should evaluate the effects of HRAM on factors associated with cartilage anabolism, such as type II collagen and aggrecan in addition to activity and/or expression of enzymes which regulate homeostasis of cartilage matrix.

We did not observe any effect of HRAM on the ratio of live- and dead-cell staining, supporting the safety of this material on chondrocyte viability at the doses tested. Viability was affected, however, by the highest dose of CM_sim_; thus provision of RA via HRAM appears to be a safer vehicle for delivery of RA to cartilage tissue than CM, presumably due to the inordinate up-scaling of other phytochemicals present in CM.

## Conclusions

It is concluded that simulated digestion in the presence of liver microsomes is an effective means for producing a biological extract of bioactive plant materials. HRAM is a strong inhibitor of PGE_2 _and NO in LPS-stimulated cartilage explants, and is a weak inhibitor of GAG release. Further research into the *in vivo *effects of this plant is warranted.

## Competing interests

The authors declare that they have no competing interests.

## Authors' contributions

WP carried out all cartilage explants experiments, conducted all biological and statistical analyses and drafted the manuscript. RSF analyzed plant material for rosmarinic acid and metabolites. LSK conceived the study and participated in its design. MBH participated in design and coordination of the study. All authors read and approved the final manuscript.

## Pre-publication history

The pre-publication history for this paper can be accessed here:

http://www.biomedcentral.com/1472-6882/10/19/prepub

## Supplementary Material

Additional file 1 Table: Effects of 24-hour LPS treatment [0 or 3 μg/mL] on explants conditioned with CM_sim_, RA, CO, CA, and FA, and unconditioned control explants. Click here for file
