# Supplementary material for: Protection against LPS-induced cartilage inflammation and degradation provided by a biological extract of Mentha spicata
Source: BMC Complement Altern Med. 2010 May 11;10:19. doi: 10.1186/1472-6882-10-19 (PMC2874512; doi:10.1186/1472-6882-10-19)
Supplement: Additional file 1 — Table: Effects of 24-hour LPS treatment [0 or 3 μg/mL] on explants conditioned with CMsim, RA, CO, CA, and FA, and unconditioned control explants. [file 1472-6882-10-19-S1.DOC]

**Table 2 – Effects of 24-hour LPS treatment (0 or 3 µg/mL) on explants conditioned with CM_sim_, RA, CO, CA, and FA, and unconditioned control explants.**

Letters represents values significantly (p<0.05) different from unconditioned controls under the same stimulatory conditions.

| **Treatment** | **Dose (µg/mL)** | **N** | **PGE_2_ (pg/mL)** | | | **GAG (µg/mL)** | | | **NO (µg/mL)** | | **C-AM:EthD-1** | |
| --- | --- | --- | --- | --- | --- | --- | --- | --- | --- | --- | --- | --- |
|  |  |  | LPS Dose (*µg/mL*) | | | LPS Dose (*µg/mL*) | | | LPS Dose (*µg/mL*) | | LPS Dose (*µg/mL*) | |
|  |  |  | 0 | | 3 | 0 | | 3 | 0 | 3 | 0 | 3 |
| **Unconditioned control** | 0.00 | **6** | 189.9 ± 5.6 | | 838.3 ± 201.8 | 86.4 ± 12.9 | 156.2 ± 28.0 | | 0.43 ± 0.07 | 1.09 ± 0.29 | 337.5 ± 21.8 | 334.9 ± 39.7 |
| **RA** | 0.34 | **6** | 174.6 ± 3.9 | | 745.1 ± 180.6 | 87.7 ± 11.2 | 106.2^a^ ± 10.4 | | 0.65 ± 0.14 | 1.00 ± 0.12 | 305.5 ± 28.5 | 250.9 ± 15.5 |
| **CO** | 0.032 | **6** | 192.7 ± 7.5 | | 874.1 ± 234.2 | 76.6 ± 19.0 | 129.6 ± 12.7 | | 0.47 ± 0.10 | 1.12 ± 0.16 | 263.2 ± 19.6^a^ | 226.7 ± 17.3 |
| **FA** | 0.023 | **6** | 203.1 ± 8.1 | | 862.2 ± 179.0 | 78.9 ± 17.7 | 123.7 ± 14.0 | | 0.54 ± 0.16 | 1.16 ± 0.11 | 284.5 ± 24.7 | 263.7 ± 17.0 |
| **CA** | 0.227 | **6** | 190.0 ± 6.4 | | 984.0 ± 371.5 | 91.9 ± 16.5 | 125.2 ± 15.5 | | 0.59 ± 0.12 | 1.15 ± 0.12 | 288.9 ± 22.0 | 255.0 ± 19.5 |
| **CM_sim_** | 144 | **6** | 166.6 ± 6.8 | | 961.6 ± 270.1 | 78.8 ± 12.4 | 125.5 ± 12.0 | | 0.37 ± 0.08 | 0.91 ± 0.18 | 310.3 ± 29.9 | 299.8 ± 42.6 |
|  | 720 | **6** | 160.9 ± 3.8 | 667.9 ± 138.5 | | 76.6 ± 14.7 | 144.1 ± 16.0 | | 0.32 ± 0.10 | 1.00 ± 0.07 | 318.9 ± 27.4 | 303.1 ± 17.1 |
|  | 1440 | **6** | 179.2 ± 3.1 | 667.9 ± 138.5 | | 101.4 ± 14.6 | 137.3 ± 9.6 | | 0.54 ± 0.12 | 1.57 ± 0.41 | 298.5 ± 32.4 | 249.8 ± 20.9 |
